# Cardiac telocytes — their junctions and functional implications

**DOI:** 10.1007/s00441-012-1333-8

**Published:** 2012-02-21

**Authors:** Mihaela Gherghiceanu, Laurentiu M. Popescu

**Affiliations:** Electron Microscopy Laboratory and Department of Advanced Studies, ‘Victor Babeş’ National Institute of Pathology, Bucharest, Romania

**Keywords:** Telocytes, Cardiomyocytes, Stem cells, Heart regeneration, Schwann cells, Fibroblasts

## Abstract

Telocytes (TCs) form a cardiac network of interstitial cells. Our previous studies have shown that TCs are involved in heterocellular contacts with cardiomyocytes and cardiac stem/progenitor cells. In addition, TCs frequently establish ‘stromal synapses’ with several types of immunoreactive cells in various organs (www.telocytes.com). Using electron microscopy (EM) and electron microscope tomography (ET), we further investigated the interstitial cell network of TCs and found that TCs form ‘atypical’ junctions with virtually all types of cells in the human heart. EM and ET showed different junction types connecting TCs in a network (*puncta adhaerentia minima, processus adhaerentes* and *manubria adhaerentia*). The connections between TCs and cardiomyocytes are ‘dot’ junctions with nanocontacts or asymmetric junctions. Junctions between stem cells and TCs are either ‘stromal synapses’ or adhaerens junctions. An unexpected finding was that TCs have direct cell–cell (nano)contacts with Schwann cells, endothelial cells and pericytes. Therefore, ultrastructural analysis proved that the cardiac TC network could integrate the overall ‘information’ from vascular system (endothelial cells and pericytes), nervous system (Schwann cells), immune system (macrophages, mast cells), interstitium (fibroblasts, extracellular matrix), stem cells/progenitors and working cardiomyocytes. Generally, heterocellular contacts occur by means of minute junctions (*point contacts*, *nanocontacts* and *planar contacts*) and the mean intermembrane distance is within the macromolecular interaction range (10–30 nm). In conclusion, TCs make a network in the myocardial interstitium, which is involved in the long-distance intercellular signaling coordination. This integrated interstitial system appears to be composed of large homotropic zones (TC–TC junctions) and limited (distinct) heterotropic zones (heterocellular junctions of TCs).

## Introduction

A telocyte (TC) is a unique type of interstitial cell with specific prolongations named telopodes (Tp) (Popescu and Faussone-Pellegrini 2010; Popescu 2011; Faussone-Pellegrini and Popescu 2011). TCs have been described by electron microscopy in several cavitary and non-cavitary organs of humans and mammalians [see www.telocytes.com]. Tp are an alternation of thin segments (podomers) and dilated segments (podoms). Podomers are very thin (less than 0.2 μm), often below the resolving power of light microscopy, explaining the fact that TCs have been overlooked up to now. In the heart, TCs have been found in the myocardium, epicardium, endocardium and cardiac stem cell niches (Popescu and Faussone-Pellegrini 2010; Li et al. 2010; Bani et al. 2010; Faussone-Pellegrini and Bani 2010; Gherghiceanu et al. 2010; Gherghiceanu and Popescu 2010; Kostin 2010; Suciu et al. 2010a; Zhou et al. 2010; Popescu et al. 2010, 2011a, b; Faussone-Pellegrini and Popescu 2011; Popescu 2011; Rusu et al. 2011) and various roles of TCs in cardiac physiology and pathology have been discussed (Mandache et al. 2010; Rupp et al. 2010; Limana et al. 2011; Ardeleanu and Bussolati 2011; Barile and Lionetti 2012; Kostin 2011; Liehn et al. 2011; Lionetti 2011; Liu et al. 2011; Manole et al. 2011; Russell et al. 2011; Sassoli et al. 2011; Xiao et al. 2011 Zheng et al. 2011; Zhou and Pu 2011; Suciu et al. 2011; Laflamme and Murry 2011). In 1963, Farquhar and Palade discovered and classified the ‘classical’ cell–cell junctions, using electron microscopy. For a long time they were considered static structures based on their conspicuous ultrastructure. However, new techniques have revealed that junctional molecules are not restricted to a particular type of junction (Franke 2009; Pieperhoff et al. 2010). Atypical homocellular junctions with discrete ultrastructure and specific molecular composition have been described in addition to the four major “textbook categories” of cell–cell junctions (gap junctions, tight junctions, adherens junctions and desmosomes) (see for review Franke et al. 2009). Anyway, a broad range of other junctions exists such as the tiny *puncta adhaerentia minima*, *manubria adhaerentia*, *plakophilin-2-containing adhaerens junctions*, etc. (Wuchter et al. 2007; Franke et al. 2009; Barth et al. 2009). Cell–cell interactions play a key role in tissue architecture as well as in cell growth, renewal, repair and pathology (Sheikh et al. 2009; Cavey and Lecuit 2009; Li and Radice 2010; Green et al. 2010; Palatinus et al. 2010; Li et al. 2011; Raju et al. 2011). In the adult mouse heart, we have found that TCs form an interstitial network connected by *homocellular* junctions and that they are also involved in formation of *heterocellular* contacts with cardiomyocytes (Mandache et al. 2007; Gherghiceanu and Popescu 2011) or cardiac stem/progenitor cells (Popescu et al. 2009; Gherghiceanu and Popescu 2010). Electron microscopy has also shown that TCs frequently establish close contacts (*stromal synapses*; Popescu et al. 2005) with several types of immunoreactive cells in various organs (Suciu et al. 2010b; Hinescu et al. 2011; Popescu et al. 2011b, Nicolescu and Popescu 2012; Nicolescu et al. 2012; Rusu et al. 2012; Cretoiu et al. 2012). We have further investigated the interstitial TC network in the human heart and have found that TCs can form ‘atypical’ junctions with virtually all types of cardiac cells.

## Material and methods

Small human heart samples (atrial appendages) were obtained from patients undergoing heart surgery. Mouse heart samples were obtained from four 1-year-old C57BL/6 mice.

**Transmission electron microscopy (EM)** was performed on cardiac samples processed according to a routine fixation and Epon embedding procedure, as previously described (Mandache et al. 2007; Hinescu et al. 2011). Thin sections (60 nm) were examined under a Morgagni 286 transmission microscope (FEI Company, Eindhoven, The Netherlands) at 60 kV. Digital electron micrographs were recorded with MegaView III charge-coupled device (CCD) using iTEM SIS software (Olympus, Soft Imaging System, Münster, Germany). All measurements were performed with iTEM SIS software, using 50 randomly selected structures/images. Several EM images were digitally colored (blue) using Adobe Photoshop CS3, in order to highlight the presence of TCs.

**Electron microscope tomography (ET)** was performed by using a Tecnai G2 Spirit BioTwin transmission electron microscope with single-tilt specimen holder (FEI Company) at 100 kV as previously described (Gherghiceanu and Popescu 2007). Electron tomographic data sets were recorded with a MegaView G2 CCD camera (Olympus) in ET mode on 250 nm-thick sections of Epon-embedded mouse cardiac tissue. Tomographs were acquired at 1-degree angular increments from −65° to +65° with an axis perpendicular to the optical axis of the microscope, at a magnification of 36,000× magnifications (1.64 nm/px). After data alignment, the data sets were reconstructed into a three-dimensional (3D) volume (data collection, reconstruction and visualization) by using Xplore3D Tomography Suite software (FEI Company). Amira 5.0.1 software (Visage Imaging, Berlin, Germany) was used for 3D imaging.

## Results

Telocytes (TCs) are clearly defined by their ultrastructural features: interstitial cells with extremely long prolongations named telopodes (Tp). The shortest definition of TCs is: cell with Tp (Popescu 2011).

Telopodes (Tp) have particular characteristics and limitations (Figs. 1, 2, 3, 4, 5, 6, 7, 8, 9, 10 and 11):

**Fig. 1:**
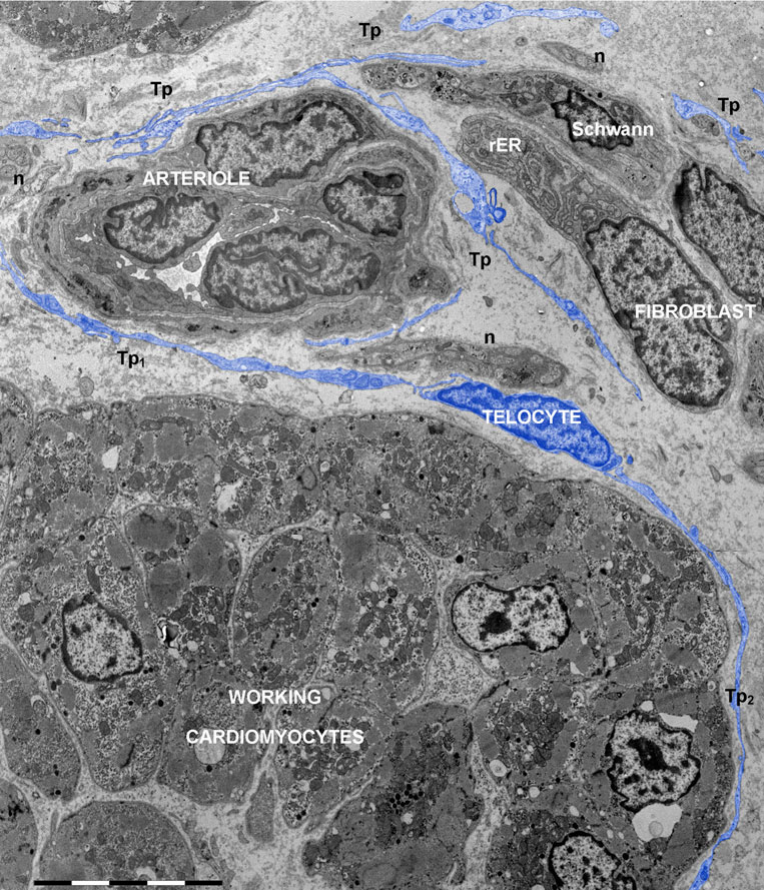
Electron micrograph of human atrium shows the interstitial network of telocytes and their telopodes (digitally colored in *blue*). Many different types of nonmyocytes are present in cardiac interstitium: telocyte (about 50 μm long), fibroblast, blood vessel, Schwann cell and numerous nerve endings (*n*). Telopodes (*Tp*) of different telocytes are visible among the interstitial cells. Telopodes *Tp*_*1*_ and *Tp*_*2*_ enfold a group of working cardiomyocytes. The fibroblast (about 15 μm long) has the cytoplasm filled with rough endoplasmic reticulum (*rER*). *Bar* 10 μm

**Fig. 2:**
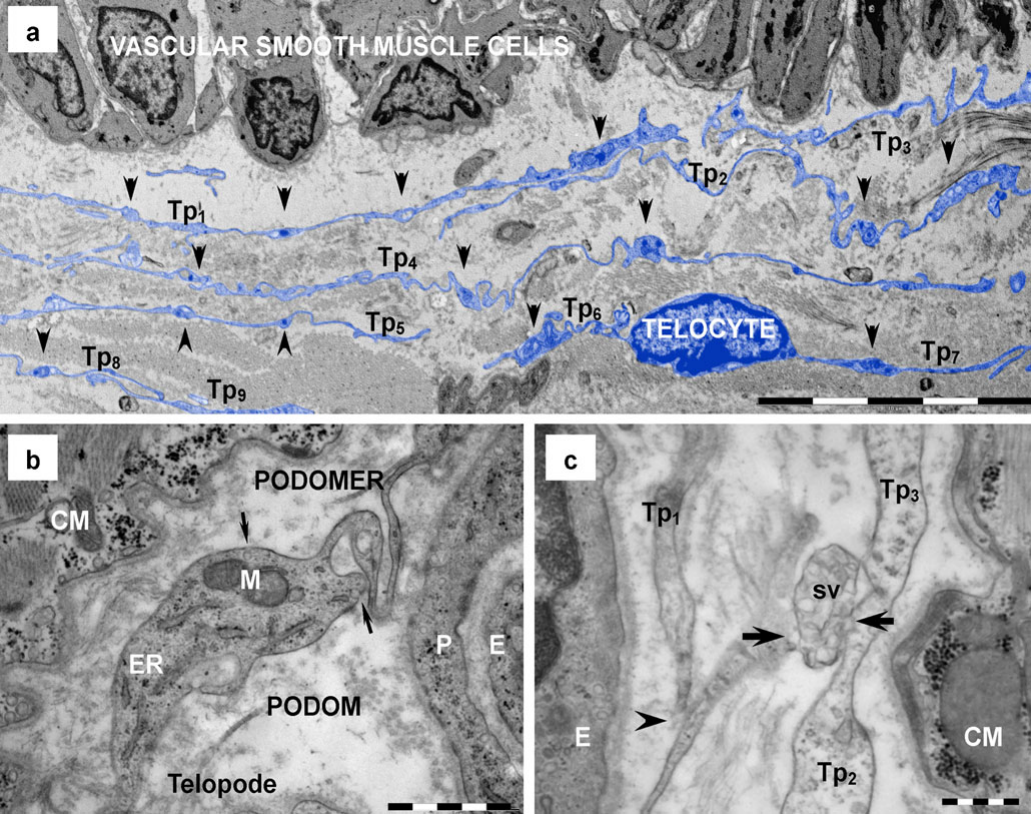
Telocytes in human heart (electron microscopy). **a** Digitally colored image emphasizes in *blue* a network of telopodes (*Tp*_*1*_ *÷Tp*_*9*_) neighboring a cardiac artery. Overlapping and parallel running telopodes are formed by alternation of podomers (less than 0.2 μm thin segments) and podoms (*arrowheads*), which generate their moniliform aspect. **b** Podoms, the dilated segments of telopodes, host mitochondria (*M*), endoplasmic reticulum (*ER*) and caveolae (*arrows*). **c** Shed vesicles (*sv*), clustered in multivesicular structures, emerge (*arrows*) from telopodes (*Tp*). The image suggests that shed vesicles (*sv*) are transferred from *Tp*_*2*_ to *Tp*_*3*_. A point contact (*arrowhead*) is visible between *Tp*_*1*_ to *Tp*_*2*_. *CM* — cardiomyocyte; *E* — endothelial cell; *P* — pericyte. *Bars* 10 μm (**a**), 1 μm (**b**), 0.5 μm (**c**)

**Fig. 3:**
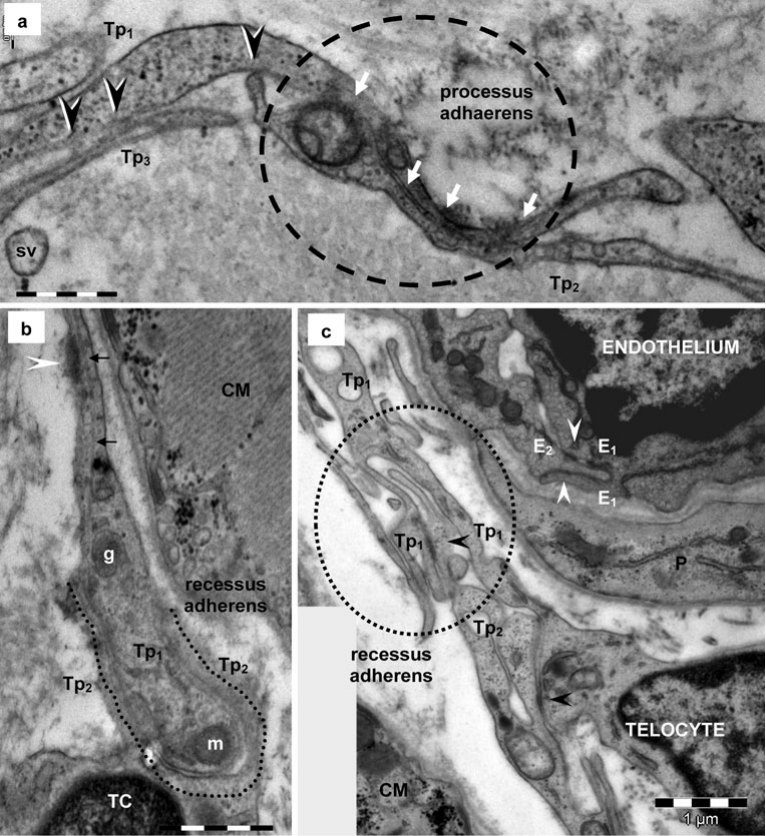
Telocyte–telocyte junctions in human heart (electron microscopy). **a** Two overlapping telopodes (*Tp*_*1*_ *, Tp*_*2*_) are connected by a sequence of *puncta adherentia minima* (*small arrows*) in 1.2 μm long contact sector (*white arrows in dotted circle*) — *processus adhaerens*. Minute adjoining points of the plasma membrane of telopodes (*Tp*_*1*_ *-Tp*_*2*_ and *Tp*_*1*_ *-Tp*_*3*_) are also visible (*black arrowheads*). **b** A telopode (*Tp*_*1*_) is embraced by the cytoplasmic extension (*Tp*_*2*_) of a telocyte (*TC*) — *recessus adhaerens* (intercellular contact is 2.7 μm long and the mean intermembrane distance is 25 nm). Note the mitochondria (m), dense core granule (g) and microtubule (*small arrows*) in telopode *Tp*_*1*_. An attachment plaque (*white arrowhead*) connects *Tp*_*1*_ with the extracellular matrix. **c** A telopode (*Tp*_*2*_) inserts into a cuplike space (*dotted circle*) formed by the adjacent telopode (*Tp*_*1*_) — a loose *recessus adhaerens* (intercellular contact is 3 μm long and the intermembrane distance is between 25 and 100 nm). Focal adherens junctions (*black arrowheads*) could be seen connecting telopodes in the junctional structure. Similar junctional construct (*white arrowhead*) can be usually seen connecting adjacent endothelial cells (*E1, E2*)*. CM* — cardiomyocyte; *P* — pericyte. *Bars* 0.5 μm (**a**, **b**), 1 μm (**c**)

**Fig. 4:**
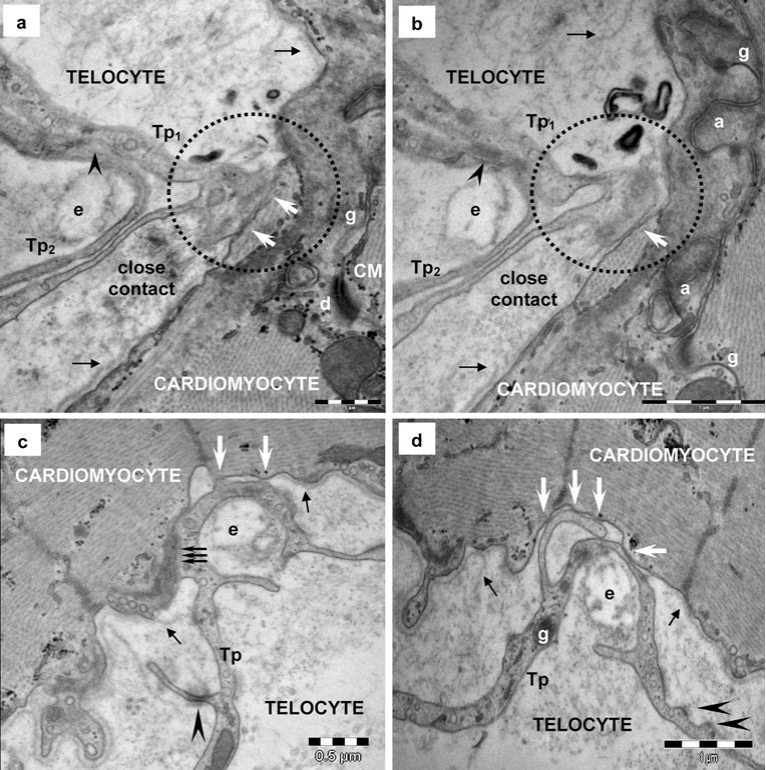
Telocyte–cardiomyocyte junctions in human heart (electron microscopy). **a**, **b** Serial sections display a tight contact (*white arrows in dotted circles*) between plasma membranes of a telopode (*Tp*_*1*_) and a cardiomyocyte. Note the discontinuity (*small black arrows*) of CM’s basal lamina. Another telopode (*Tp2*) makes planar contact (*arrowheads*) with Tp_1_ and wraps an elastic fiber (*e*). A desmosome (*d*), gap (*g*) and adherens (*a*) junctions are visible connecting the cardiomyocytes. **c**, **d** Serial ultrathin sections additionally show ‘atypical’ junction connecting a telopode (*Tp*) and a cardiomyocyte (*CM*). The junction is formed by small point contacts (*white arrows*) apparently randomly distributed. *Triple arrows* point out a connection segment where the telopode and cardiomyocyte seem to fuse (**c**). Microfilaments form a cytoplasmic plaque in the cardiomyocyte cortical space at the site of asymmetric junction. Basal lamina of the cardiomyocyte is interrupted on this segment and *small black arrows* mark the break points. Note that the telopode (*Tp*) makes a loop around an elastin fiber (*e*). Attachment plaques (*arrowheads*) connect the telopode with the extracellular matrix. A dense core granule could be seen in the telopode in panels **d** (*g*). *Bars* 0.5 μm (**a**, **c**), 1 μm (**b**, **d**)

**Fig. 5:**
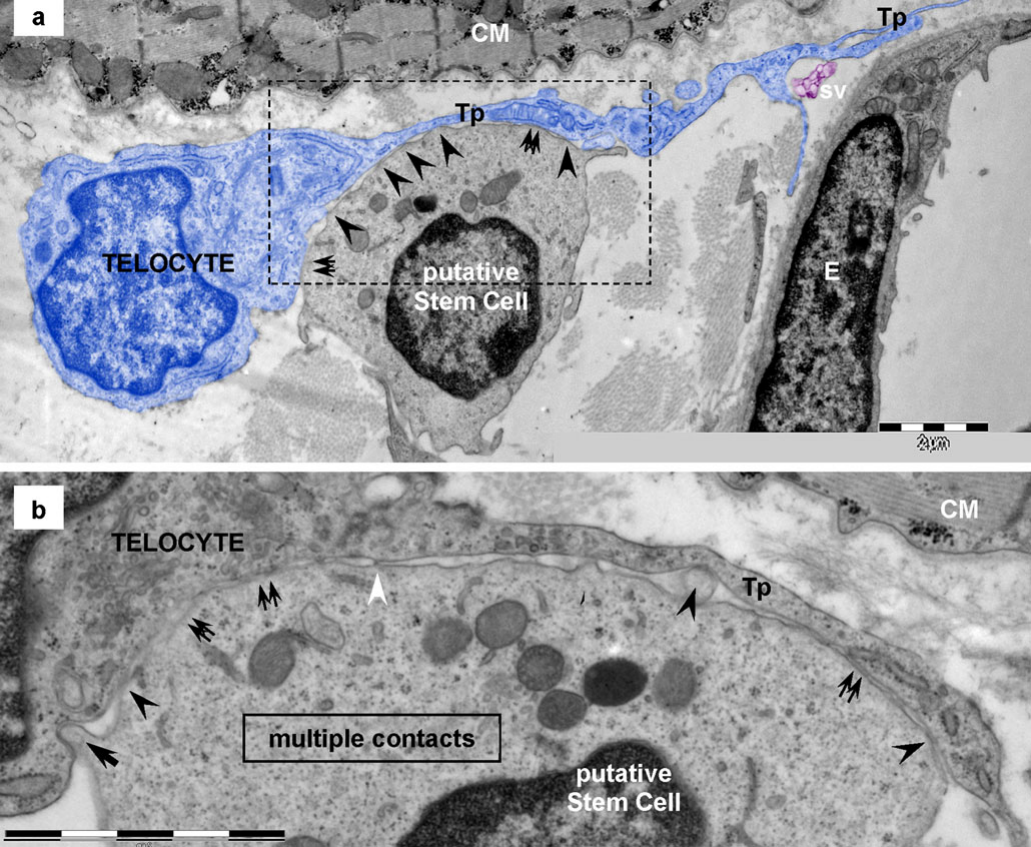
Telocyte–putative stem cell junctions in human heart (electron microscopy). **a**, **b** Electron microscopy shows the point contacts (*arrowheads*) between a telocyte (*blue colored*) and a putative stem cell. Broader, planar contacts (*double arrows*) could also be seen. **a** The mean distance between plasma membranes of telopode (*Tp*) and putative stem cell is 43 ± 20.3 nm (min: 20.3 nm; max: 90.6 nm). *CM* — cardiomyocyte; *sv* — shed vesicles; *E* — endothelial cell. **b** Higher magnification on a consecutive ultrathin section of the *rectangular area* marked in **a** highlights the geometry of the 8-μm-long heterocellular connection: dot contacts (*arrowheads*) alternate with planar contacts, tight-fitting apposed sectors of plasma membranes (*double arrows*). Small cellular projection of putative stem cell (*arrow*) inserts into a small recess of the telocyte. Dense nanostructures (15–20nm) could be seen connecting the plasma membranes of the two cells (*white arrowheads*). *Bars* 2 μm (**a**, **b**)

**Fig. 6:**
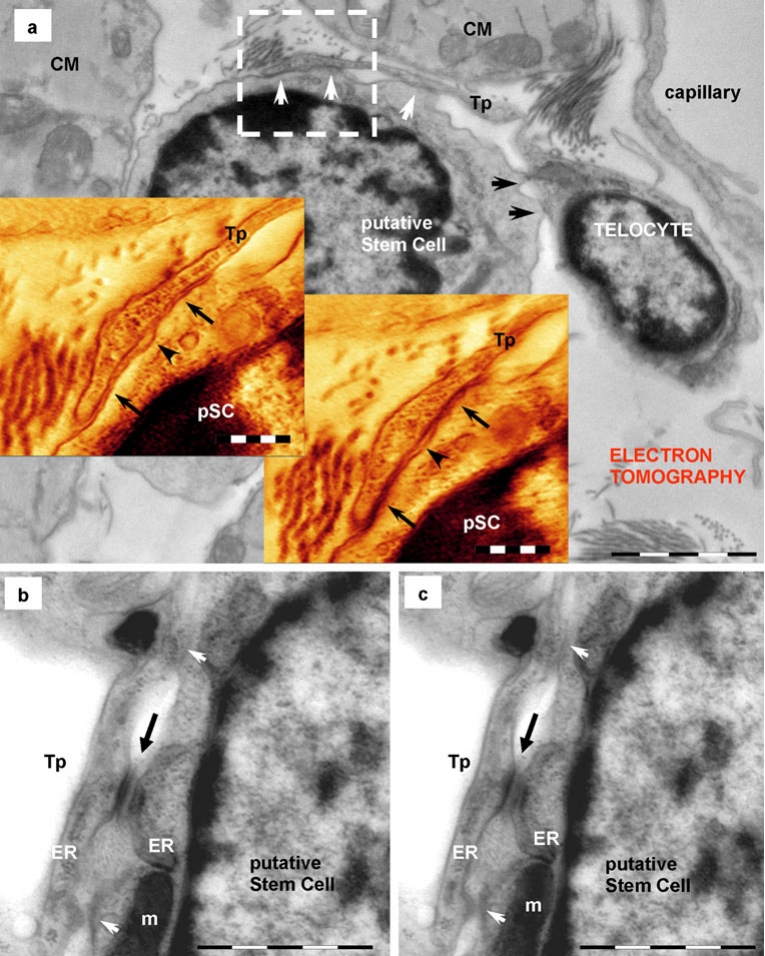
Telocyte–putative stem cell junctions (*pSC*) (electron tomography). Telocyte via telopode (*Tp*) makes heterocellular and heterotypical junctions with a putative stem cell in a mouse cardiac stem cell niche. **a** The image in the background (direct image of the 250-nm-thick section) shows the overall appearance of the multiple contacts: planar contacts (*white arrows*) and point contacts (*black arrows*). The *two insets* show digital sections (60 and 81 from 89) from the reconstructed volume of *square marked area*. *Arrows* mark planar contacts between telopode (*Tp*) and putative stem cell (pSC). A small space (*arrowheads*) is delimited by the two planar contacts. **b**, **c** Digital sections through another tomographic volume show adherens junction (*black arrows*) and lateral point contacts (*white arrows*) between a telopode (*Tp*) and a putative stem cell. Endoplasmic reticulum cisternae (*ER*) are visible in both cells. *m* — mitochondrion. *Bars* 2 μm (**a**), 0.5 μm (*insets* in **a**), 1 μm (**b**, **c**)

**Fig. 7:**
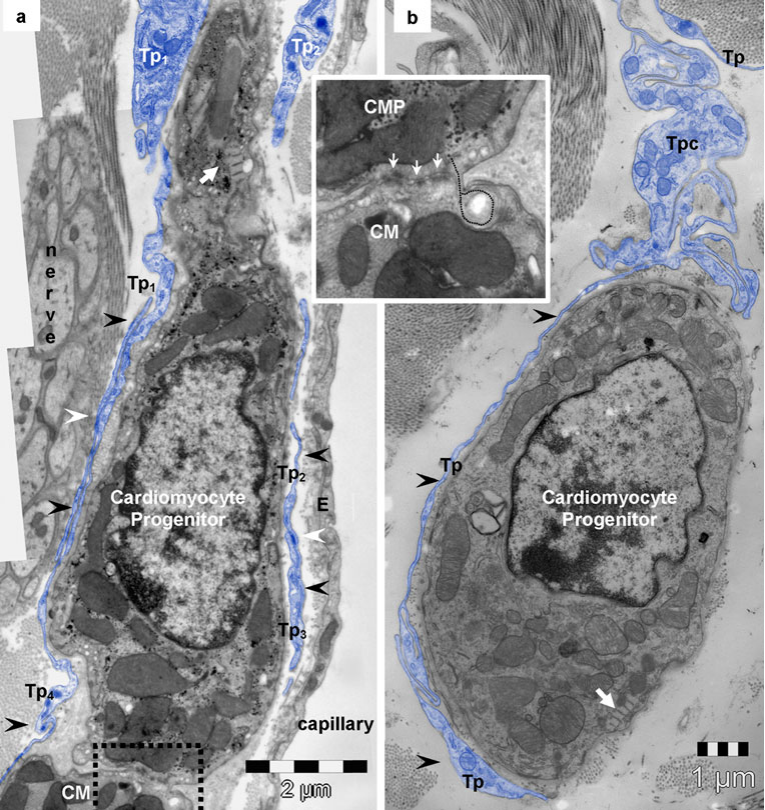
Telocyte–cardiomyocyte progenitors (*CMP*). **a**, **b** Electron microscopy images of mouse heart show telopodes (*Tp, blue*) surrounding cardiomyocyte progenitors (*arrowheads*) in the stem cell niche. The intercellular, intermembrane, distance is below 150 nm. *White arrows* indicate typical organelles for CMP-leptofibrils. **a** *White arrowheads* point out small adherens junctions between overlapping telopodes (*Tp*_*1*_ with *Tp*_*4*_ *; Tp*_*2*_ with *Tp*_*3*_) embracing CMP. *Rectangular marked area* (details in *inset*) highlights how CMP adjoin in the periphery or cardiac muscle (CM). *Inset* — higher magnification reveals immature adherens junctions (*white arrows*) fastening *CMP* addition to the working cardiomyocyte (*CM*). *Dotted line* follows the insertion of a small process of CMP into a recess of the adult CM. **b** Note the convoluted segment of the telopode (*Tpc*) above the CMP

**Fig. 8:**
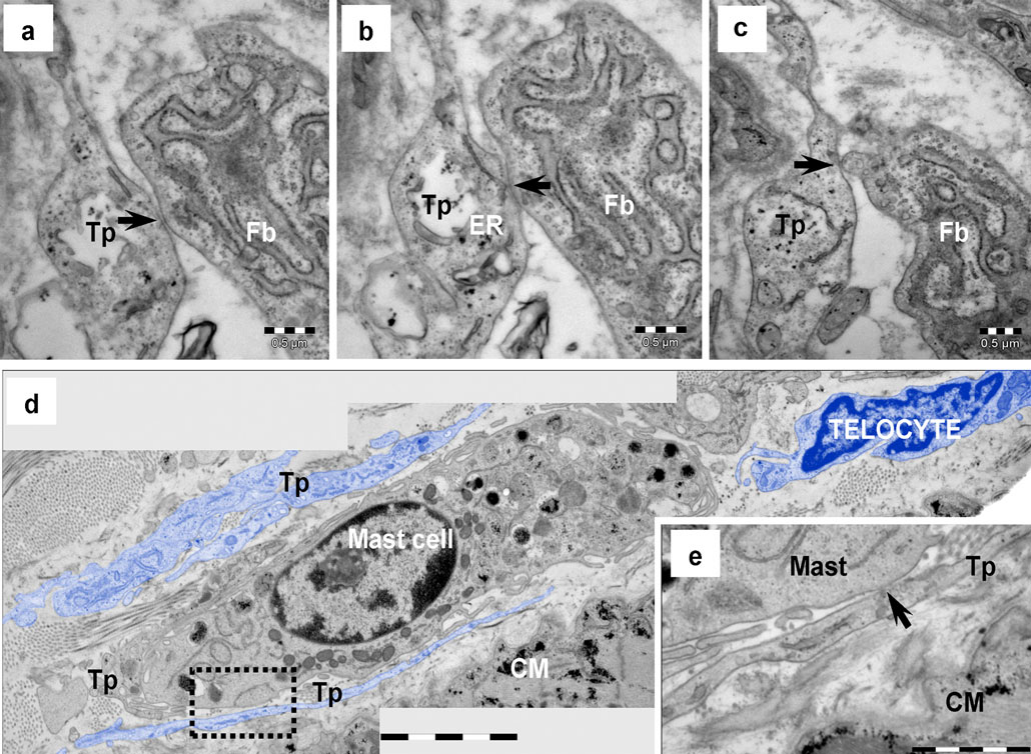
Electron microscopy of human heart demonstrates the existence of atypical junctions between the telocyte and fibroblast (**a**–**c**) as well as between the telocyte and mast cell (**d**, **e**). **a**–**c** Serial ultrathin sections illustrate the telocyte–fibroblast connection. Electron-dense nanostructures (*arrows*) could be observed connecting a telopode (*Tp*) with a fibroblast (*Fb*). **d** A mast cell is surrounded by telopodes (*Tp, blue colored*). **e** High magnification of *squared marked area* in **e** shows, on a consecutive ultrathin section, electron-dense nanostructures (*arrow*) connecting the telopode (*Tp*) with the mast cell. *CM* — cardiomyocytes. *Bars* 0.5 μm (**a**–**c**, **e**), 2 μm (**d**)

**Fig. 9:**
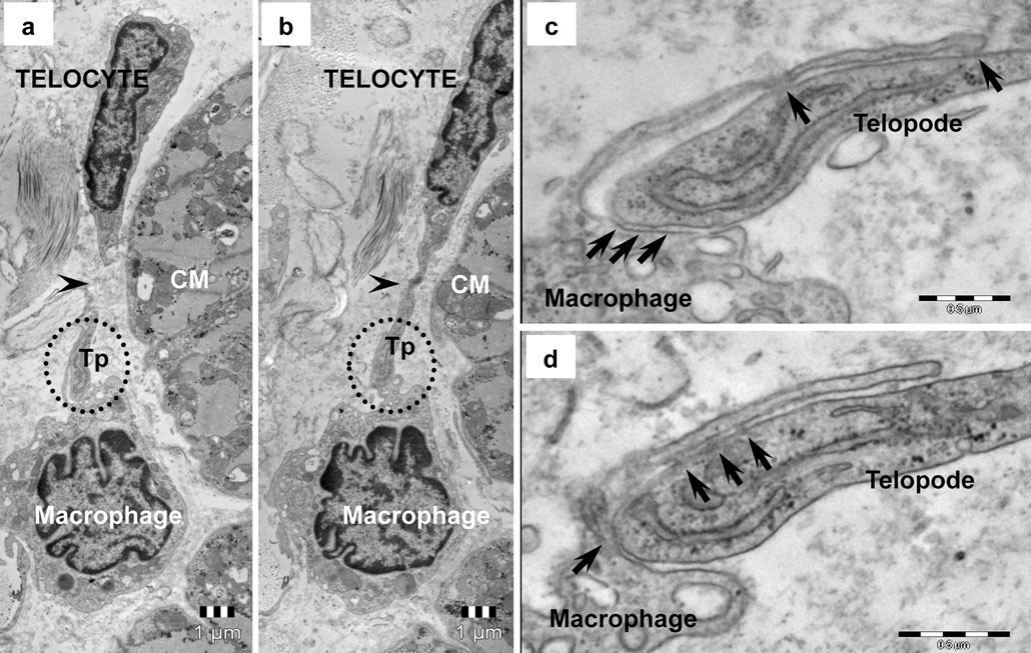
Telocyte–macrophage junction in human heart (electron microscopy). **a**,**b** Serial ultrathin sections illustrate the discontinuity of the telopode (*Tp, arrowheads*) attributable to its sinuous path. **c**,**d** High magnification of *round marked areas* in **a** and **b** shows on serial sections, apparently random distributed electron-dense nanostructures (*arrows*) connecting the telopode and slim process of the macrophage

**Fig. 10:**
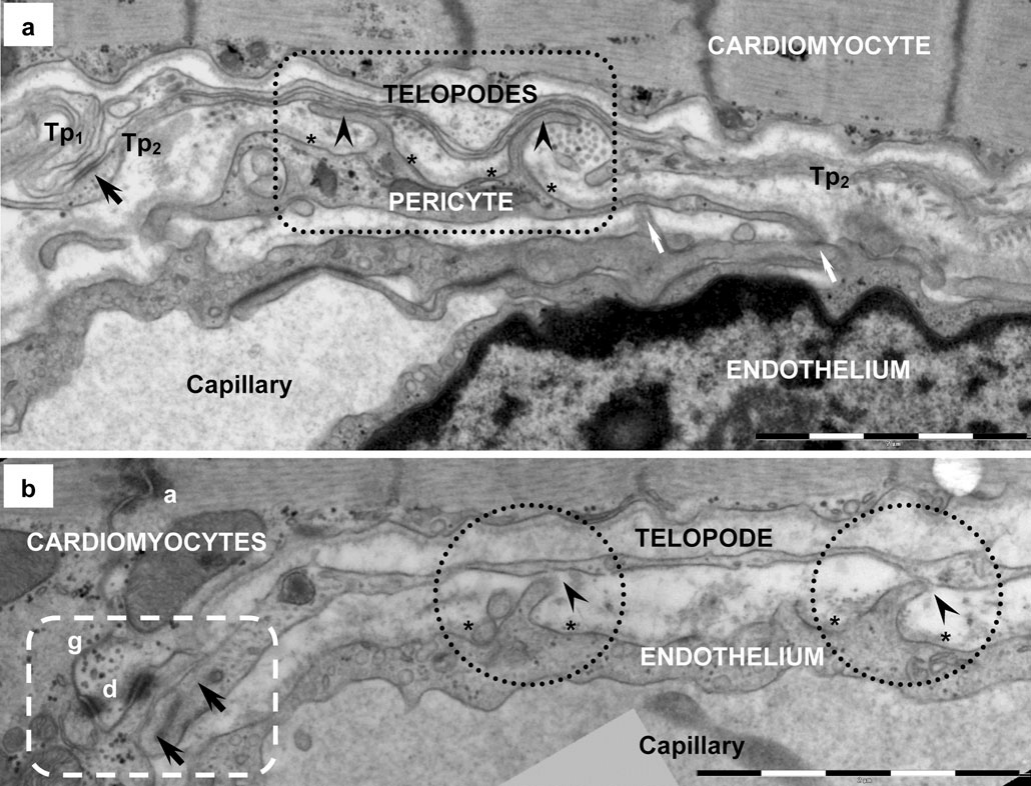
Telocyte–capillary junction in human heart (electron microscopy). **a** Two overlapping telopodes (*Tp*_*1*_ *, Tp*_*2*_), connected by plaque-bearing *puncta adhaerentia* junction (*black arrow*), are positioned between a cardiomyocyte and a capillary. Two tight contacts (*arrowheads in rectangle mark*) are noticeable between the pericyte and telopode *Tp*_*2*_. Basal lamina of the pericyte is broken up (*asterisks*) by two short processes extending to *Tp*_*2*_. *White arrows* point out tight contacts between the endothelial cell and the pericyte (myoendothelial junctions). **b** A telopode (*Tp*) has two point contacts (*arrowheads in dotted circles*) with the endothelial cell. Basal lamina (*asterisks*) of the endothelium is interrupted and short processes of the endothelial cell extend toward the *Tp*, comparable to the myoendothelial junctions in panel **a**. *Rectangular dotted mark* surrounds the additional heterocellular junction (*arrows*) between the same telopode (*Tp*) and the cardiomyocyte. Basal lamina of cardiomyocytes is discontinuous at the junctional site. Desmosome (*d*), gap (*g*) and adherens (*a*) junctions are visible in the intercalated disk. A dense core granule is visible in the telopode, nearby the heterocellular junctional complex. *Bars* 2 μm (**a**, **b**)

**Fig. 11:**
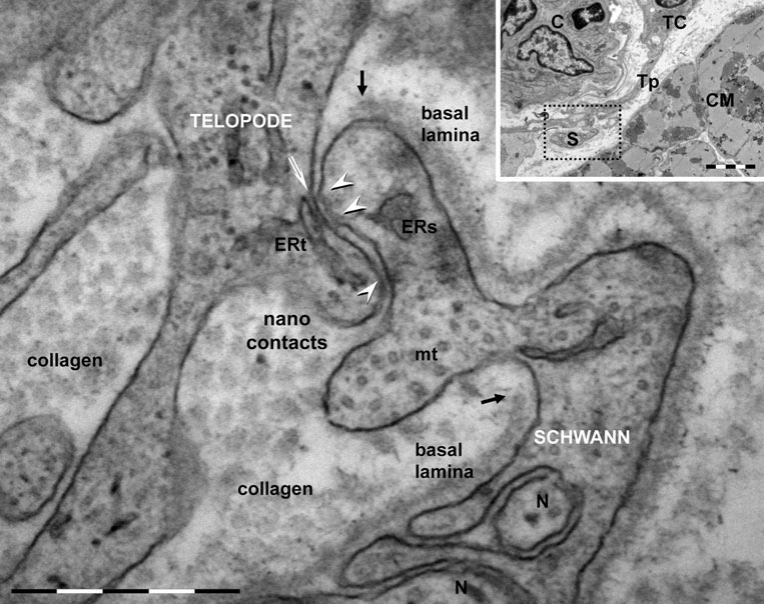
Telocyte–Schwann cell junction in human heart (electron microscopy) formed by point contacts. About 10 nm electron-dense nanostructures (nanocontacts) are visible bridging plasma membranes of the TC’s telopode and Schwann cell (*arrowheads*). The basal lamina of Schwann cell is broken up (*black arrows*) lateral to the junctional site. Endoplasmic reticulum (*ERs*) and microtubules (*mt*) are visible in Schwann cell, which enfolds nerve endings (*N*). Endoplasmic reticulum (*ERt*) in telopode is connected by dense nanostructures or ‘feet’ (*white arrow*) with plasma membrane fronting the junction. The *inset* shows the overall image: *TC* — telocyte; *Tp* — telopode, *S* — Schwann and nerve endings, *CM* — cardiomyocytes, *C* — capillary. *Bars* 0.5 μm, 2 μm (*inset*)

1. Number : 1–5/cell, usually 1–3 (Figs. 1 and 2);
2. Branching: dichotomous pattern (Fig. 3);
3. Length: usually tens of micrometers (up to 100) (Figs. 1 and 2);
4. Aspect: moniliform — podomers alternating with podoms (Fig. 2);
5. Podomers (Fig. 2) — 50–100 nm thin segments; usually below 0.2 μm (the resolving power of light microscopy) (116.91 ± 58.64 nm; min = 29.26 nm; max = 261.82 nm/*n* = 50);
6. Podoms (Fig. 2) — dilated segments accommodating mitochondria, ER and caveolae (‘Ca^2+^ uptake/release units’) (0.65 ± 0.23 μm; min = 0.32 μm; max = 1.19 μm/*n* = 50);
7. Connected with each other via homocellular junctions (Fig. 3) form an interstitial 3D network (Figs. 1 and 2a).

### Telocytes — homocellular junctions

One of the most striking features of TCs is their organization in a 3D network by Tp connections through homocellular junctions. Non-characteristic junctions connecting TCs usually occur at the level of Tp but junctions between the Tp and TCs cell body are also encountered (Fig. 3c). Moreover, electron microscopy often shows that Tp are connected by point contacts and electron-dense nanostructures (Fig. 3a). The two cell membranes are separated by a narrow space (10-30 nm), suggesting a molecular interaction between different TCs.

Electron microscopy showed that TCs are coupled by adherens junctions with different morphology: *puncta adhaerentia minima* (Figs. 3a, 7a and 10a), *processus adhaerentes*, visible between overlapping telopodes (Figs. 3a, 4a, b and 7a) and *recessus adhaerentes or manubria adhaerentia* (Fig. 3b, c). The *recessus adhaerentes* junctions were visible between Tp (Fig. 3c) and regions of the cellular body of TCs or between Tp segments of different cells (Fig. 3b). It is worthy of mention that no unambiguous gap junction has been found connecting TCs.

In addition to all direct membrane-membrane homocellular contacts, electron microscopy also showed that shedding vesicles (60-100 nm vesicles) and clusters of microvesicles or exosomes (diameters: 250 – 350 nm up to 1 μm) were frequently emerging from Tp (Fig. 2c). The mean diameter of shed vesicles was 128.6 ± 33.3 nm (min: 60 nm; max: 193 nm / n = 50).

### Telocytes — heterocellular junctions

Electron microscopy revealed that cardiac TCs could establish heterocellular junctions with all other cell types existing in the heart: cardiomyocytes (CM) (Figs. 4 and 10b), putative stem cells (pSC) (Figs. 5 and 6), cardiomyocyte progenitors (CMP) (Fig. 7), fibroblasts (Fig. 8a–c), mast cells (Fig. 8d, e), macrophages (Fig. 9), pericytes (Fig. 10a), endothelial cells (Fig. 10b) and Schwann cells (Fig. 11). Direct heterocellular contacts found by electron microscopy were point contacts, electron-dense nanostructures and planar contacts (Table 1). No typical ultrastructural features of ‘classical’ types of junctions have been found (gap, tight, adhaerens or desmosomes).

**Table 1:** The intermembrane distances in heterocellular junctions formed by telocytes with various cell types in adult heart

Cell type	Junction type	Rough estimation of intermembrane distance	Intermembrane distance (*n* = 50)
Working cardiomyocytes	close vicinity	<150 nm	122.79 ± 20.61 nm (min = 79.80 nm; max = 158.22 nm)
	point contacts	10–30 nm	21.36 ± 4.06 nm (min = 10.20 nm; max = 28.34 nm)
	nanocontacts	10 nm	9.98 ± 1.32 nm (min = 8.24 nm; max = 13.02 nm)
Cardiomyocyte progenitors	close vicinity	<150 nm	106.27 ± 30.09 nm (min = 77.53 nm; max = 181.75 nm)
Putative stem cells	point contacts	10–30 nm	21.66 ± 3.95 nm (min = 12.79 nm; max = 30.08 nm)
	planar contacts	10–25 nm	16.19 ± 6.46 nm (min = 9.20 nm; max = 24.48 nm)
Endothelial cells	point contacts	10–30 nm	23.55 ± 5.03 nm (min = 12.09 nm; max = 27.57 nm)
Pericytes	planar contacts	10–20 nm	16.18 ± 3.48 nm (min = 10.47 nm; max = 20.59 nm)
Schwann cells	point contacts	10–30 nm	17.12 ± 7.87 nm (min = 7.89 nm; max = 26.81 nm)
	nanocontacts	10 nm	10.45 ± 2.59 nm (min = 8.69 nm; max = 12.72 nm)
Macrophages	point contacts	10–30 nm	18.32 ± 5.03 nm (min = 12.09 nm; max = 27.57 nm)
Mast cells	point contacts	10–30 nm	24.70 ± 4.75 nm (min = 16.70 nm; max = 29.12 nm)
Fibroblasts	point contacts	10–30 nm	17.17 ± 3.04 nm (min = 13.79 nm; max = 21.63 nm)

### Telocytes — cardiomyocytes

Frequently, TCs are close to the basal lamina of cardiomyocytes and the distance between the two cellular membranes is about 150 nm. Occasionally, direct contacts between TCs and cardiomyocytes have also been observed (Figs. 4 and 10b). The basal lamina of cardiomyocytes appears to be split apart lateral to the contact sites (Fig. 4). Sometimes, EM images suggest a fusion of the cell membranes of TCs and cardiomyocytes (Fig. 4a, c) but the exploration of serial thin sections (Fig. 4b, d) shows that ‘fusion’ is a false impression generated by the picture of obliquely sectioned membranes. Direct connections TC–CM have been undoubtedly found (Fig. 4b, d), dot junctions connecting the cellular membranes. Small electron-dense nanostructures have been seen linking the cellular membranes of TCs and CMs (Fig. 4c, d). Some TC–CM junctions appear to be asymmetric. Dense material (Z-band like) could be observed in cortical cytoplasm of cardiomyocytes in some points (Fig. 4c) but no specific ultrastructure in the counterpart TC cytoplasm. The TC–CM junctions could often be observed at the level of intercalated discs (Figs. 4a, b and 10b) but TC–CM contacts could be seen at various distances from intercalated discs (Fig. 4c, d).

### Telocytes — putative stem cells

Electron microscopy showed that TCs have direct contacts with mononuclear cells, probably stem cells (Figs. 5 and 6a). These putative stem cells (pSC) are small, round-oval cells (6–10 μm in diameter), with few mitochondria, few long endoplasmic reticulum cisternae and a large amount of free ribosomes (Fig. 5). Anyway, a set of criteria for stem cell recognition by electron microscopy has already been reported (Gherghiceanu et al. 2011). Usually, TCs have small contacts with pSC (Gherghiceanu and Popescu 2010) but sometimes Tp attach to the plasma membrane of pSC and the ultrastructure of the membrane connections resemble a stromal synapse (Popescu et al. 2005) with multiple close-contact points alternating with planar direct intermembrane contacts and regions of wider intermembrane distance (50-100 nm) (Fig. 5). Serial sections show that short processes of pSC insert into small recesses of TCs and form minute ‘*recessus adhaerens*’ - like junctions (Fig. 5b). Electron tomography shows that planar contacts have small dense structures bordering on the contact membrane of pSC (Fig. 6a). In addition, typical adherens junctions could be observed (Fig. 6b, c).

### Telocytes — cardiomyocyte progenitors

Unlike stem cells, the cardiomyocyte progenitors (Popescu et al. 2009; Gherghiceanu et al. 2011) are recognizable without difficulty (Fig. 7). These cells display typical ultrastructural features of immature cardiomyocytes, including high nucleo-cytoplasmic ratios, unorganized bundles of filaments, lipid droplets, intracytoplasmatic dense bodies (similar to primordial Z lines), intracytoplasmatic desmosome-like structures (primordial intercalated discs) and cortical leptofibrils (Fig. 7). Moreover, these cells have large mitochondria, numerous caveolae and a continuous basal lamina. A central element of the niche is represented by TCs, stromal supporting cells for CMP (Popescu et al. 2009; Gherghiceanu and Popescu 2010).

### Telocytes — other interstitial cells

Point contacts or planar junctions could often be found between TCs and fibroblasts (Fig. 8a-c), mast cells (Fig. 8d, e) or macrophages (Fig. 9). Small electron dense nanostructures were usually present between plasma membranes of contacting cells (Figs. 8 and 9) but classical type of junctions has not been found.

### Telocytes — capillaries

Electron microscopy showed contacts between TCs and capillaries, in particular pericytes (Fig. 10a) and endothelial cells (Fig. 10b). There were point contacts (Fig 10b) or planar contacts (Fig 10a) but no electron dense structures were present on plasma membranes or in the cortical cytoplasm to subsume these contacts under one of the known classes of intercellular junctions. The basal lamina of both endothelial cells and pericytes was always broken up at the level of heterocellular junctions (Fig. 10). The relationships between TCs and endothelial cells (Manole et al. 2011) as well as between TCs and pericytes (Suciu et al. 2011) have previously been reported. Endothelial cells and pericytes usually establish heterotypic myocyte–endothelial junctions (Fig. 10a).

### Telocytes — Schwann cells

Our ultrastructural study showed that TCs also establish direct cell–cell point contacts with Schwann cells (Fig. 11), for example in human atrial tissue. The basal lamina of Schwann cells presented discontinuities at the site of contacts. The maximal diameter of these atypical heterocellular junctions was up to 0.5 μm. Electron dense nanostructures (about 10 nm) were usually present between plasma membranes of TCs and Schwann cells (Fig. 11). Cisternae of endoplasmic reticulum could often be seen next to junctional areas (Fig. 11).

## Discussion

We have previously reported that TCs and CMs are directly connected by small dense structures (10–15-nm nanocontacts) and suggested that TC–CM might represent a ‘functional unit’ (Gherghiceanu and Popescu 2011). The present study reveals that intercellular communication in human heart is much more complex than actually thought (see the recent viewpoint by Kohl and Camelliti 2011).

The ultrastructural analysis showed that TCs form an interstitial system that assembles all cardiac cells in an integrative network. TCs have direct cell–cell communication not only with CMs but with all interstitial cells (Table 1). Among interstitial cells, TCs seem to be particularly involved in heterocellular communication and this study endorses the idea of the TC cardiac network as structural and functional support for long-distance signaling, essential in cardiac renewing physiology (Popescu et al. 2011a).

From an ultrastructural point of view, TC–CM junctions do not fit in any acknowledged pattern — there are no specific structures to be classified in one of the known junction types, either classical (Farquhar and Palade 1963) or newly described (Franke et al. 2009). Usually, clusters of nanocontacts (‘nanofeet’) fasten the connection between TCs and CMs plasma membranes with no interposition of the basal lamina. The bridging nanostructures (about 10 nm) and the intermembrane distances (10–30 nm) essentially suggest a molecular interaction between the TC and CMs (Gherghiceanu and Popescu 2011). Using EM, we did not identify any gap junction connecting TCs and CMs, as has been reported connecting the fibroblasts and CMs (e.g., Kakkar and Lee 2010; Kohl and Camelliti 2011). The discrepancy between the results previously reported about Cx43 immunofluorescence and our EM results reported here might be explained by the fact that Cx43 is a highly regulated phosphoprotein and has a half-life of less than 2 hours (Lampe and Lau 2004). Anyway, the main (if not the only) unequivocal diagnosis for a ‘gap junction’ remains EM. In addition, we could not find any cellular fusion (Driesen et al. 2005) or nanotubules (Hurtig et al. 2010) connecting TCs and CMs. Partial heterocellular fusion has also been reported in vivo between cardiac fibroblasts and dedifferentiated CMs in the border zone of a rabbit myocardial infarction (Driesen et al. 2005) but EM images presented were not compelling. A thicker section or an oblique section though the contact area could generate a false image (see Fig. 4).

An unexpected finding was that TCs have also direct cell–cell contacts with Schwann cells. We have not found any reference about junctions between cardiac nerve endings, specifically Schwann cells, or any other interstitial cells. Recently, it was reported that signaling between fibroblasts and Schwann cells results in cell sorting, followed by directional collective cell migration of Schwann cells out of the nerve stumps to guide axons regrowing across the wound (Parrinello et al. 2010). The TCs–Schwann cell interaction should be important for cardiac renewal and regeneration. Moreover, TCs establish contacts with pericytes, or directly with endothelial cells. The EM shows that these junctions are similar with myoendothelial junction, which possibly is a cellular integration point in the vascular (patho)physiology (Heberlein et al. 2009).

The distance between TCs and other interstitial cells (macrophages, fibroblasts, mast cells) is often within the range of tens of nm (10 to 30 nm), which also fits in the macromolecular interactions domain but which molecules are involved in heterocellular communication remains to be established. Additionally, a paracrine and/or juxtacrine secretion of small molecules and long-distance signaling by shedding microvesicles may play distinct roles in horizontal transfer of important macromolecules among neighboring cells (Ramachandran and Palanisamy 2011). Shed vesicles and exosomes are molecular complex intercellular signaling organelles (involved in this acellular mode of communication) with multiple functions, which appear as promising new tools for clinical diagnostics and potentially for novel therapeutic strategies (Lee et al. 2011). TCs release shed vesicles and/or exosomes, thus sending macromolecular signals (e.g., microRNAs, Cismasiu et al. 2011) to neighbor cells and thus modifying their transcriptional activity (Barile and Lionetti 2012).

TCs seem to be active players in cardiac renewing, since they are ‘nursing’ CM progenitors in epicardial stem cell niches (Popescu et al. 2009; Gherghiceanu and Popescu 2010). Moreover, electron microscope tomography has revealed complex nanoscopic junctions between TCs and resident stem/progenitor cells. Apparently, TCs provide tracks (long telopodes) for the “evolution” (sliding) of precursor cells towards mature CMs (Fig. 7; Popescu et al. 2009) and their integration into heart architecture (Gherghiceanu and Popescu 2010). Last but not least, TCs are directly (nanocontacts) and indirectly (paracrine secretion, VEGF and NO) involved in neoangiogenesis, within the border zone of experimental myocardial infarction (Manole et al, 2011).

An enhanced understanding of the cells involved in and the signals, pathways that mediate the regenerative response may be useful in modulating the regenerative response of the injured heart. Irrespective of location, a stem cell niche capable of housing stem cells entails few constitutive elements with regulatory properties (Jones and Wagers 2008): (1) stromal supporting cells (also called nurse cells, niche cells or supporting cells) that interact directly with the stem cells and with each other, (2) extracellular matrix proteins that provide mechanical signals to the niche, (3) blood vessels that transmit systemic signals and bring circulating stem cells if needed and (4) neural inputs that might communicate distant physiological cues to the stem cell microenvironment. TCs produce an adequate microenvironment for precursor cells and guide them from epicardium into the myocardium and therefore should be considered as nurse cells (Gherghiceanu and Popescu 2010; Popescu et al. 2011a). Furthermore, this study showed that TCs cardiac network could integrate the overall ‘information’ from the vascular system (endothelial cells and pericytes), nervous system (Schwann cells), immune system (macrophages, mast cells), interstitium (fibroblasts, extracellular matrix) and working cardiomyocytes. The integration of all these heterocellular signals may be essential for the decision of stem cells (resident or exogenous) to proliferate, differentiate and mature into new CMs or other cardiac cell types.

The understanding of the interstitium as an integrating system is more sensitive nowadays when cellular therapy is a key word of science. The interstitial space seems to be the place where regenerative process happens but little is known about the cells involved and how they act together (Barile and Lionetti 2012). The structural and functional interactions between CMs and other cardiac cells are essential for understanding heart (patho)physiology and for the further development of efficient cell therapies (Kohl and Camelliti 2011). Hitherto, we have found only TCs interacting with CMs and nonmyocytes in normal heart. One key question is whether all the interstitial cells (stem/progenitor cells, macrophages, Schwann cells, fibroblasts, mast cells, etc.) make contacts with each other or with CMs. The concept of "cardiovascular unit" as a building block of the heart, which includes CMs, adjacent capillaries and fibroblasts has recently been proposed (Ausoni and Sartore 2009). Myocardial tissue functions as a well-organized community of cells and presumably the TC network is enough to offer the physical support for heterocellular communication and coordinates their individual activities.

This study shows that homotropic and heterotropic ultrastructural interactions of TCs form an integrative interstitial cardiac system, which possibly assures physiological coordination of multicellular signals, essential for cardiac renewal, regeneration and repair. Further, molecular analysis must identify the key players involved in the telocytes communication network and their role in cardiac physiology and pathology.
